# A review of mixed malaria species infections in anopheline mosquitoes

**DOI:** 10.1186/1475-2875-10-253

**Published:** 2011-08-31

**Authors:** Mallika Imwong, Supatchara Nakeesathit, Nicholas PJ Day, Nicholas J White

**Affiliations:** 1 Department of Molecular Tropical Medicine and Genetics, Faculty of Tropical Medicine, Mahidol University, Bangkok, Thailand; 2 Center for Emerging and Neglected Infectious Diseases, Mahidol University, Bangkok, Thailand; 3 Mahidol Oxford Research Unit, Faculty of Tropical Medicine, Mahidol University, Bangkok, Thailand; 4 Centre for Tropical Medicine, Churchill Hospital, Oxford, UK

## Abstract

**Background:**

In patients with malaria mixed species infections are common and under reported. In PCR studies conducted in Asia mixed infection rates often exceed 20%. In South-East Asia, approximately one third of patients treated for falciparum malaria experience a subsequent *Plasmodium vivax *infection with a time interval suggesting relapse. It is uncertain whether the two infections are acquired simultaneously or separately. To determine whether mixed species infections in humans are derived from mainly from simultaneous or separate mosquito inoculations the literature on malaria species infection in wild captured anopheline mosquitoes was reviewed.

**Methods:**

The biomedical literature was searched for studies of malaria infection and species identification in trapped wild mosquitoes and artificially infected mosquitoes. The study location and year, collection methods, mosquito species, number of specimens, parasite stage examined (oocysts or sporozoites), and the methods of parasite detection and speciation were tabulated. The entomological results in South East Asia were compared with mixed infection rates documented in patients in clinical studies.

**Results:**

In total 63 studies were identified. Individual anopheline mosquitoes were examined for different malaria species in 28 of these. There were 14 studies from Africa; four with species evaluations in individual captured mosquitoes (SEICM). One study, from Ghana, identified a single mixed infection. No mixed infections were identified in Central and South America (seven studies, two SEICM). 42 studies were conducted in Asia and Oceania (11 from Thailand; 27 SEICM). The proportion of anophelines infected with *Plasmodium falciparum *parasites only was 0.51% (95% CI: 0.44 to 0.57%), for *P. vivax *only was 0.26% (95% CI: 0.21 to 0.30%), and for mixed *P. falciparum *and *P. vivax *infections was 0.036% (95% CI: 0.016 to 0.056%). The proportion of mixed infections in mosquitoes was significantly higher than expected by chance (P < 0.001), but was one fifth of that sufficient to explain the high rates of clinical mixed infections by simultaneous inoculation.

**Conclusions:**

There are relatively few data on mixed infection rates in mosquitoes from Africa. Mixed species malaria infections may be acquired by simultaneous inoculation of sporozoites from multiply infected anopheline mosquitoes but this is relatively unusual. In South East Asia, where *P. vivax *infection follows *P. falciparum *malaria in one third of cases, the available entomological information suggests that the majority of these mixed species malaria infections are acquired from separate inoculations.

## Background

Where transmission of both vivax and falciparum malaria is high in parts of Oceania, mixed species infections in humans are common. With frequent infection from repeated inoculation it is not surprising that infections accumulate and so comprise a multiplicity of genotypes and species. In South-East Asia where transmission of malaria is low, seasonal, and unstable people typically receive one infected mosquito bite each year or less, yet a remarkably high proportion (30 to 50%) of acute *Plasmodium falciparum *malaria infections are followed soon afterwards by an infection with *Plasmodium vivax*. Indeed in endemic areas in this region, the main clinical complication of falciparum malaria is subsequent vivax malaria, far outweighing the incidence of recrudescent falciparum malaria [1-5]. The intervals between presentation with falciparum malaria and the subsequent vivax episode are very similar to the intervals between acute vivax malaria and the first relapse [2,5]. This suggests that the vivax malaria episode, which follows falciparum malaria, also results from a relapse. The very high rate of co-infection in South-East Asia has suggested that the two infections are acquired together despite the low transmission frequency.

Mixed species infections in patients are often under reported, with a tendency to over report the more dangerous *P. falciparum*. In endemic areas, where the prevalence of malaria revealed by sensitive PCR methods is much higher than evident from microscopy, high rates of mixed blood stage infection (~20%) have been reported, presumably because both infections are carried chronically [6,7]. Lower rates of mixed species infection occur in symptomatic non-immunes presenting with acute falciparum malaria in low transmission settings. Even with sensitive PCR detection of low parasitaemia, the proportion of mixed infections detected does not approach the 30 to 50% required to explain the proportion of vivax malaria episodes, which follow *P. falciparum *malaria in these same patients. Simultaneous infection studies in the malaria therapy of neurosyphilis and in volunteers clearly showed that mixed infection could occur from simultaneous inoculation, that pre-erythrocytic development of *P. falciparum *was more rapid, and that in the blood stage infection *P. falciparum *tended to suppress *P. vivax*, so low level *P. vivax *parasitaemia might still be missed by current diagnostic methods [8-10]. *Plasmodium vivax *may also suppress *P. falciparum*; up to 10% of acute vivax malaria episodes in Thailand are followed shortly after by *P. falciparum *infections without reinfection [3,11]. Blood stage infections in which both parasites are detectable could either derive from simultaneous inoculation of the sporozoites of the two species from a doubly infected anopheline mosquito, or a recently acquired infection of one species might supervene a chronic infection with the other. As transmission intensities in most of South East Asia are very low (EIRs typically < 1/year) the probabilities of separate inoculations within a narrow time window are extremely low [12]. To resolve these questions and understand better the epidemiology of mixed species infection in different transmission settings we examined the published literature on immunological and molecular parasite species identification in anopheline vectors in malaria endemic areas.

## Methods

The NLM PubMed was searched since 1987 for publications in English describing studies on anopheline vectors in malaria endemic areas in which malaria parasite identification had been performed using immunological or molecular methods. The search terms were; anopheles AND sporozoite OR oocyst; Subheadings: Asia OR Africa OR America. Studies of both trapped wild mosquitoes and artificially infected mosquitoes were included. After compilation the lists were checked manually. The location of study and year, the collection method, mosquito species, number of specimens, parasite stage examined (oocysts or sporozoites), parasite detection method, and the results of the species identification were tabulated. Investigators in the South East Asian region were contacted for further information. ELISA and more recently PCR methods were used generally for species identification. The sensitivity of these different methods were also reviewed.

## Results

In total, 42 studies were identified which were conducted in Asia and Oceania (11 were from Thailand) and 21 studies were conducted in other areas (14 were from Africa and 7 from Central or South America) between 1987 and 2011. Three of the American and three of the Asian studies involved blood feeding of anophelines and later dissection. In the remainder of the studies trapped wild anophelines were examined.

### Sensitivity of different methods used for parasite identification

Table 1 shows the different methods used to identify mixed infections and, where quoted, their respective sensitivities and limits of detection.

**Table 1:** Sensitivity of different methods of parasite detection used in studies of mixed infection

	Detection method	References	Species	Gold standard	Sensitivity	Specificity	Quoted lower limit
1	CS-ELISA	Wirtz et al.,1985 [17]	PV-VK210 PV-VK247				125-250 sporozoites per 30 μL mosquito extract

2	CS-ELISA	Wirtz et al.,1987 [18]	PF				100 sporozoites per mosquito

3	CS-ELISA	Collins et al.,1988 [19]	PM				25-50 sporozoites per mosquito

4	PCR on *pfdhfr-ts*	Harada et al.,2000 [20]	PF				4 pg of DNA (100 P/μL)

5	PCR on ssrRNA	Snounou et al.,1993 [21,22]	PF, PV, PM, PO				10 P/μL

6	Semi-nested PCR on ssrRNA	Larduex et al.,2008 [23]	PF, PV, PM, PO				3 sporozoites (0.06 pg of DNA)

		Rubio et al.,1999 and 2002 [24,25]		Thick- thin films	100%	67%	0.1 P/μL

7	PCR-RFLP on Cyt B gene	Hasan et al,2009 [26]	PF, PV-VK210, PV-VK247				0.2 pg of DNA

8	Real-time PCR ssrRNA	Mangold et al.,2005 [27]	PF, PV, PM, PO	Microscopy analysis	PF 94.1% PV100% PO 100%	PF100% PV99.1% PO100%	0.01-0.02% parasitaemia

9	RT-PCR on *pfg377 *and *pfs16 *mRNA	Nakazawa et al.,2009 [28]	PF				1 parasite per salivary gland

10	Real-time PCR 18S rRNA	Perandin et al.,2004 [29]	PF, PV, PO	Nested-PCR	100%	100%	PF 0.7P/μL, PV 4P/μL, PO 1.5P/μL

11	Dipstick	Bangs et al.,2002 [30]	PF, PV-VK210, PV-VK247	CS-ELISA	100%	97%	-

12	Vectest Dipstick	Ryan et al.,2002 [31]	PF, PV-VK210, PV-VK247	CS-ELISA	92%	98.10%	-

13	Fluorescein-labelled DNA probe	Lulu et al.,1997 [32]	PF				500 sporozoites

14	Microtiter plate hybridization	Kimura et al.,1997 [33] Tangin et al.,2008 [34]	PF, PV, PM, PO				0.0001-0.0005% parasitaemia

P: malaria parasite, CS: circumsporozoite protein, *pfdhfr-ts*: *Plasmodium falciparum *dihydrofolate reductase-thymidylate synthase. PF: *Plasmodium falciparum*, PV: *Plasmodium vivax*, PO: *Plasmodium ovale*, PM: *Plasmodium malariae*.

### Entomological studies from Asia and Oceania (Table 2, and 3)

**Table 2:** Studies from Asia

References	Countries	No. with positive sporozoites																					
		**PF**	**Total**	**PV**	**Total**	**VK210**	**Total**	**VK247**	**Total**	**PM**	**Total**	**VK210+VK247**	**Total**	**PF+PVVK210**	**Total**	**PF+PVVK247**	**Total**	**PF+PM**	**Total**	**PF+PV+PK**	**Total**	**malaria+**	**Total**

**ASIA**																							

Upatham et al., 1988 [35]	Thailand	2	111																				

Coleman RE et al., 2002 [36]	Thailand	4	8452			8	8452	3	8452			2	8452										

Bangs MJ et al., 1996 [37]	Irian Jaya, Indonesia	5	2518			8	2518	3	2518	2	2518			1	2518								

Frances SP 1996 [38]	Thailand	1	276					2	42			2	54										

Nakazawa S 2009 [28]	Vietnam																	1	30	1	30		

Mya MM 2002 [39]	Myanmar	2	212																				

Gingrich JB 1990 [12]	Thailand	17	2247	12	2247																		

Harbach 1987 [40]	Thailand	2	463	4	590																		

Toma T 2002 [41]	Khammouane, Lao	1	63	1	13																		

Oh SS et al., 2010 [42]	Ganghwa-do (Island), Korea			2	1845																		

Dev et al., 2010 [43]	North-east India																					2	88

Sahu SS et al., 2008 [44]	Orissa State, India																					13	1178

Lee HW et al., 2002 [45]	Korea					4	4870	3	4870														

Mahapatra N et al., 2006 [46]	Orissa State, India	7	204																				

Hasan et al.,2009 [26]	Papua New Guinea	1	107																				

Cooper et al.,2009 [47]	Papua New Guinea	106	22970			11	22970	43	22970	10	22970					4	22970						

Bortel et al.,2010 [48]	Ninh Thuan, Vietnam	9	6540			2	6540	5	4047														

Mohanty et al.,2009 [49]	Orissa, India	45	692																				

Burkot et al.,1989 [50]	Madang Province, Papua New Guinea																					484	83442

Burkot et al.,1992 [51]	Madang Province, Papua New Guinea	72	14777			56	14777	16	14777	1	14777			5	14777								

Bockarie et al.,1996 [52]	Sepik province, Papua New Guinea	76	4168	43	4168																		

Hii et al.,2000 [53]	Sepik province, Papua New Guinea	691	102996			108	102996	197	102996	83	102996												

Writz et al,1987 [54]	Madang, Papua New Guinea	10	669	13	669																	26	651

Total		**1051**	**167465**	**75**	**9532**	**197**	**163123**	**272**	**160672**	**96**	**143261**	**4**	**8506**	**6**	**17295**	**4**	**22970**	**1**	**30**	**1**	**30**	**525**	**85359**

**Table 3:** Studies from Asia

References	Countries	No. with positive oocysts						No. with positive whole mosquitoes											
		**PF**	**Total**	**PV**	**Total**	**PF+PV**	**Total**	**PF**	**Total**	**PV**	**Total**	**PV-VK210**	**Total**	**PV-VK247**	**Total**	**PF+PV**	**Total**	**VK210+VK247**	**Total**

**ASIA**																			

Toma T 2002 [41]	Khammouane, Lao			1	10	1	391												

Alam et al., 2010 [55]	Bangladesh							8	302			7	306	1	7				

Trung et al., 2004 [56]	Vietnam, Cambodia							14	892			1	28	2	361			2	550

Tangin et al., 2008 [34]	Bangladesh							12	669	1	669					6	669		

Swain et al.,2009 [57]	Orissa, India							2	186										

**Total**		**0**	**0**	**1**	**10**	**1**	**391**	**36**	**2049**	**1**	**669**	**8**	**334**	**3**	**368**	**6**	**669**	**2**	**550**

**Figure 1:**
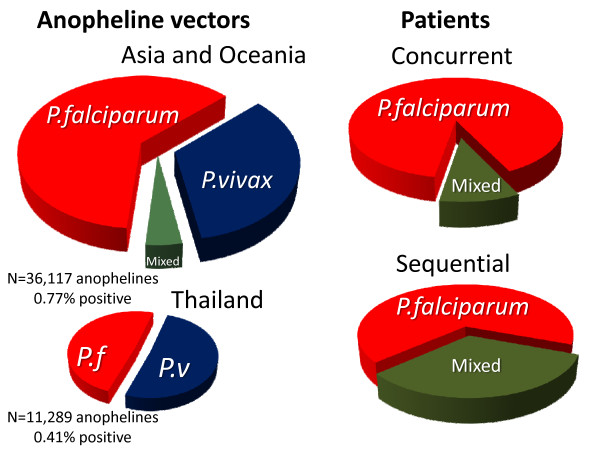
**Left side: Pie charts showing the proportion of individual anopheline vector mosquitoes examined which were positive for *P. falciparum, P. vivax *or both malaria parasite species in entomological studies conducted in Asia and Oceania (top) and in the subgroup of studies conducted in Thailand (bottom)**. On the right side are pie charts showing the proportion of malaria patients in Thailand with *P. falciparum, P. vivax *or both malaria parasite species concurrently assessed by PCR typing of blood samples (top). The proportion of patients in Thailand who have *P. vivax *infections following *P. falciparum *(without the possibility of reinfection) within a nine week period are shown below.

In four studies, sporozoites were examined but not speciated; 525 of 85,359 mosquitoes were positive. In ten studies some or all of the anophelines caught were pooled in groups before analysis. In these ten studies 155,497 anophelines were examined for *P. falciparum *and 184,744 were examined for *P. vivax*. Only one study reported mixed infections but, because of pooling, it cannot be ascertained whether this was in single mosquitoes. In 27 studies of trapped wild mosquitoes 167,465 individual anophelines were examined for *P. falciparum *sporozoites and in a further 2,049 the whole body was assessed. *P. falciparum *parasites only were identified in 1087 (0.51%; 95% CI: 0.44 to 0.57%). For *P. vivax *334,698 anophelines were assessed and 544 were positive (0.25%%; 95% CI: 0.21 to 0.30%) for *P. vivax *alone. Mixed *P. falciparum *and *P. vivax *infections were assessed in 41,325 mosquitoes. If there was no association between the parasites then none of these would have been expected to have mixed species infection, whereas 17 (0.036%: 95% CI: 0.016 to 0.056%) had evidence of infection with both parasites (p < 0.001) (Figure 1). In studies conducted in Thailand, where most of the clinical trial information on mixed species infections has come from, 11,289 anophelines were examined in published studies and 23 *P. falciparum *(0.20%) and 24 *P. vivax *(0.21%) infections, but no mixed *P. falciparum *and *P. vivax *infections were found (Figure 1). *Plasmodium malariae *was found in 97 of 143,261 anophelines examined (0.051%), one of which was also infected with *P. falciparum*. Only one of the *P. malariae *was from mainland SE Asia. One mixed *P. falciparum, P. vivax *and *Plasmodium knowlesi *infection was identified in Vietnam. No *Plasmodium ovale *was identified. The VK210 and 247 *P. vivax *genotypes were distinguished in most studies; there were 207 VK210 and 279 VK247, and six were mixed genotype infections. None of the four artificial feeding studies in which different vectors were fed on parasitaemic blood yielded any mixed infections despite high rates of positivity for the individual parasite species.

### Comparison of mixed species infection rates in patients and in the mosquito vectors in Asia and Oceania

Studies conducted on the treatment of falciparum malaria during and immediately after the first world war indicated that vivax malaria commonly followed falciparum malaria acquired in Europe or Asia. In India rates ranged from seven to 40% [13]. Between March 1924 and July 1925 Sinton compared two different quinine regimens in the treatment of falciparum malaria in British soldiers [13]. They were followed for eight weeks at Kasauli (above the level of malaria transmission at an altitude of 6,000 feet in Himachal Pradesh). Of 76 soldiers completing follow-up 30 (39%) had subsequent vivax malaria. More recent information has come mainly from South-East Asia. In 1987, Looareesuwan *et al *[1] reported that of 320 adult patients treated in the Bangkok Hospital for Tropical Diseases and followed in hospital so that reinfection could be excluded definitely, 104 (32%) had a subsequent recurrence of vivax within two months. The relapse rate for vivax malaria in the same institution was 50% (200/400) [14].

Douglas *et al *[4] recently reviewed treatment responses in 10,549 patients (4,960 children aged < 15 years and 5,589 adults) treated for falciparum malaria in a malaria endemic area on the NorthWest border of Thailand. Reinfection could not be excluded. Of these patients, 9,385 (89.0%) had *P. falciparum *monoinfection and 1,164 (11.0%) had mixed *P. falciparum/P. vivax *infections according to microscopy at presentation. The cumulative proportion with *P. vivax *recurrence by day 63 was very similar to that documented in Bangkok; 31.5% (95% CI, 30.1% to 33.0%). However the overall rate of *P. vivax *infection following *P. falciparum *monoinfection may have been underestimated as the cumulative risk of vivax malaria was 51.1% (95% CI, 46.1% to 56.2%) after treatment with rapidly eliminated drugs (t_1/2_,1 day), 35.3% (95% CI, 31.8% to 39.0%) after treatment with intermediate half-life drugs (t_1/2 _1 to 7 days), and 19.6% (95% CI, 18.1% to 21.3%) after treatment with slowly eliminated drugs (t_1/2 _> 7 days). Some late relapses suppressed by the slowly eliminated drugs may therefore have emerged after day 63. The cumulative 63 day relapse/recurrence rate for vivax malaria at the same location was 63% (143/227)[15]. Two entomological evaluations were performed in this location. Of 596 anopheline vectors (*Anopheles dirus, Anopheles minimus, Anopheles maculatus*) examined individually five carried *P. vivax *and two carried *P. falciparum*. There were no mixed infections (McGready R: personal communication).

Smithuis *et al *[5] recently studied therapeutic responses to different ACT regimens in Myanmar in 808 adults and children with acute falciparum malaria or mixed species infections (16%). 330 (41%) patients had one or more episodes of *P. vivax *infection during follow-up. Of the 679 patients with *P. falciparum *infections only, 235 (35%) had subsequent *P. vivax *malaria. Children were nearly three times more likely to develop intercurrent vivax malaria than adults.

Taking the more conservative data from Bangkok in Thailand where reinfection could be excluded, then if all vivax infections are acquired from simultaneous inoculation it would be expected that the mixed falciparum and vivax infection proportion in mosquitoes would be 32% of all the *P. falciparum *infected mosquitoes

$$\text{i}.\text{e}.\;\;0.32=\left[\text{Mixed Pf}+\text{Pv}\right]∕\left(\text{P}.\text{ falciparum}+\left[\text{Mixed Pf}+\text{Pv}\right]\right)$$

In fact no mixed species infections were recorded in Thailand among 23 *P. falciparum *positive mosquitoes (Figure 1), whereas seven or eight would have been expected if all mixed species infections had been acquired by simultaneous inoculation (p = 0.01). Overall in Asia and Oceania only 6.6% of the *P. falciparum *infected mosquitoes also carried *P. vivax*, a proportion that is considerably lower than expected from the clinical data.

### Entomological studies from Central and South America (Table 4)

**Table 4:** Studies from Central and South America, and Africa

References	Countries	No. with positive sporozoites												No. with positive sporozoites		No. with positive whole mosquitoes			
		**PF**	**Total**	**PV**	**Total**	**VK210**	**Total**	**VK247**	**Total**	**PM**	**Total**	**PO**	**Total**	**PF+PM**	**Total**	**PF**	**Total**	**PV-VK210**	**Total**

**OUTSIDE ASIA**																			

Lulu et al., 1997 [32]	Ethiopia	4	198																

Appawu et al., 2003 [58]	Ghana (KND)	929	7635																

Moreno et al., 2004 [59]	Equatorial Guinea	38	214																

Bigoga et al., 2007 [60]	Cameroon	131	1596							11	1391								

Oyewole et al., 2006 [61]	southwestern Nigeria															32	702		

Cuamba et al., 2006 [62]	Angola	12	707																

Quiñones et al., 2006 [63]	Southern Colombia																	36	775

dos Santos et al., 2009 [64]	Mapuera, Brazil	3	6350			11	6350	3	6350	1	412								

Kasili et al., 2009 [65]	Kibera slum of Nairobi, Kenya	0	80																

Taye et al., 2006 [66]	Sille, southern Ethiopia	4	796	14	796														

Annan et al.,2007 [67]	Cameroon, Kenya	221	10296																

Abonusum et al.,2011 [68]	Ghana	106	139							7	122	7	122	1	122				

Tanga et al.,2011 [69]	Cameroon	612	9940																

Shililu et al.,1998 [70]	Mumias, Kakamega, Kenya	151	2517																

**Total**		2211	40468	14	796	11	6350	3	6350	19	1925	7	122	1	122	32	702	36	775

In five of the seven studies identified some or all the mosquitoes were pooled before analysis and in three studies anopheline mosquitoes were infected artificially. One of these studies examined only for *P. vivax*. None of these studies detected mixed infections despite estimated infection rates in the pooled mosquitoes ranging from 0.1 to 3.2% for *P. vivax *and 0 to 0.87% for *P. falciparum*. In the two studies in which individual anophelines were examined (one from Columbia, the other from Brazil) 7,125 individual anophelines were examined. *P. falciparum *sporozoites only were identified in 3 (0.042%), *P. malariae *in 1 (0.014%), and 50 were positive (0.70%) for *P. vivax *alone (47 Pv VK 210 and 3 Pv VK 247). No mixed *P. falciparum *and *P. vivax *infections were identified.

### Entomological studies from Africa (Table 4)

In one of the 14 studies identified mosquitoes were artificially infected and in two studies mosquitoes were pooled before analysis. Eight studies examined the mosquitoes only for *P. falciparum*. In the artificial feeding study conducted in Guinea-Bissau where all species were investigated 510 anophelines were examined and infection rates with single species ranged from 5 to 15% for *P. falciparum*, 1 to 2% for *P. malariae *and 0.54 to 5% for *P. ovale*. Mixed infection rates ranged from 0.4 to 15%. In the 11 studies in which individual anophelines were examined 1654 of 27738 (6%) had *P. falciparum *sporozoites although rates for individual vectors in different studies varied between 0.7% and 78%. In the four studies where mixed infections were studied specifically, two studies examined only for *P. vivax*. In the remaining two studies one from Cameroon did not identify mixed infections. In the other study from Ghana overall infectivity rate was 7.7% for *Anopheles gambiae *(s.s) and 4.9% for *Anopheles funestus*. Of the sporozoite positive mosquitoes 5.7% contained *P. malariae *only and 5.7% contained *P. ovale *only and a single *A. gambiae *(s.s) was identified carrying mixed *P. falciparum *and *P. malariae*. The remainder were positive for *P. falciparum *only. No mixed *P. falciparum *and *P. vivax *infections were identified. *P. vivax *was reported only from Ethiopia where 14 (1.8%) of 796 mosquitoes were positive for *P. vivax *and 4 (0.5%) were positive for *P. falciparum*. No mixed infections were identified in this study.

## Discussion

In areas where malaria transmission is stable and intense the majority of the human population is constantly infected, although older children and adults often have parasite densities below the level of microscopy detection. Many parts of Africa have this pattern of malaria transmission. Mixed genotype *P. falciparum *infections are common, but there are relatively few studies which have examined the prevalence of other infections. In Asia and Oceania, these areas of intense transmission tend to be very small focal forest or forest fringe areas, and much of the coastal part of the Island of New Guinea. In those areas where both *P. falciparum *and *P. vivax *are prevalent mixed species infections with both species are common but maybe also be below the level of microscopy detection [8]. Both infections coexist chronically [16]. Sensitive PCR methods reveal high rates of mixed species infection (~20%) [6] although it seems likely that the majority of mixed infections in humans are acquired from separate mosquito inoculations. Asymptomatic or oligosymptomatic individuals who are parasitaemic have declared themselves able to control the infections and so are much more likely to have a multiplicity of different genotypes present (albeit at low densities) and also mixed species infections.

In contrast in much of the remainder of Asia transmission is generally low and seasonal and, although *P. vivax *infections may still be asymptomatic, *P. falciparum *malaria is usually accompanied by illness and treatment seeking. Symptomatic infections are seen at all ages, although the burden of symptomatic vivax malaria is in young children. In this epidemiological context mixed species infections are found in 10% or less of acute infections, yet approximately one third of patients still experience vivax malaria following falciparum malaria. The high efficacy of initial falciparum malaria treatment against *P. vivax *and the time interval between initial presentation and the subsequent vivax malaria episode suggests that it is a relapse. This presumed relapse was thought to result from simultaneously acquired mixed species infection, but the evidence reviewed here does not support this hypothesis. The proportion of *P. falciparum *infected mosquitoes overall which also carried *P. vivax *in Asia and Oceania was only 6.6% overall. As expected most mixed infections in mosquitoes were found in studies on the island of New Guinea where transmission of both species is intense. None were found in Thailand where one third of *P. falciparum *malaria is followed by *P. vivax*. Thus, the proportion of mixed infections in anopheline mosquito vectors in Asia and Oceania is approximately one fifth of that expected. These findings suggest that simultaneous inoculation of the two malaria species does occur, and indeed is more frequent than would be expected by chance alone, but that the majority of mixed species infections do not derive from a single mosquito bite.

Several explanations are possible. The entomology sampling frameworks in these studies may not have been representative. The vectors might not have been adequately representative of those transmitting malaria. The laboratory methods may have been insensitive (Table 1) or not designed specifically to identify mixed species infections. These concerns were addressed to some extent in those studies in which some mixed infections were identified. Lack of sensitivity seems an unlikely explanation for the observations. As only a small fraction of the salivary gland sporozoites are inoculated by a biting anopheline mosquito- it seems unlikely that these would derive from very small subpopulations of different species sporozoites which were below the level of antigen or PCR detection.

If the mixed species infections are all acquired from simultaneous inoculation it would also requires very high rates of concomitant *P. falciparum *and *P. vivax *gametocyte carriage at infectious densities in the blood of the parasitaemic population. This may well occur in high transmission settings but is rare in low unstable transmission settings and is not supported by the mosquito feeding studies reported here. As *P. vivax *develops more rapidly than *P. falciparum *in the anopheline vector, it also demands mosquito longevity. If the two infections were acquired separately then the close and reproducible temporal association between the infections needs explanation. Although the distribution of infected mosquito biting undoubtedly clusters it would require a remarkable degree of association for one third of all *P. falciparum *inoculations to be accompanied within a day or two by a *P. vivax *inoculation -when average inoculation rates are less than one infected bite per person per year! Nevertheless mixed species infections in anopheline mosquitoes do occur in Asia significantly more frequently than expected by chance so it is clear that simultaneous transmission does occur and does contribute to mixed infections. It is likely to account for the significant proportion of acute symptomatic malaria in low transmission settings where both species are identified in the blood at presentation.

The data from Central and South America where transmission is generally low and unstable also showed low rates of mosquito infection. No mixed infections were found but as *P. falciparum *and *P. malariae *infection rates were also very low this negative finding has limited significance. There are surprisingly few data from Africa on species other than *P. falciparum. Plasmodium vivax *was identified in Ethiopia as expected. In Ghana where transmission is intense mixed infection was identified. Artificial infection studies in Guinea-Bissau suggested that mixed infection of mosquitoes would be frequent, but these data are too few to draw firm conclusions on the entomology of mixed malaria infection in Africa.

This review is almost certainly an underestimate of the available information. For example, much of the Central and South American literature is in Spanish or Portuguese and some of the African literature is in French. The search strategy employed may well have missed studies in English, which lacked the key words. Nevertheless because of the large apparent discrepancies between mixed species infection rates in the anopheline mosquito vectors and humans this comprehensive sample of the literature provides a convincing message. Mixed infections of anopheline vectors do occur and account for some of the mixed infections in humans. However in South East Asia where *P. vivax *infections follows *P. falciparum *malaria in one third of cases, the published data suggests that the majority of these mixed infections are not acquired by simultaneous inoculation. A more plausible explanation is that *P. vivax *relapse is stimulated by symptomatic falciparum malaria infection.

## Competing interests

The authors declare that they have no competing interests.

## Authors' contributions

NJW, MI and NJPD conceived of this review. MI and SN carried out the review. NJW and NJPD conducted the analysis. MI, SN, NJPD and NJW participated in the interpretation and presentation. MI and NJW drafted the manuscript. All authors read and approved the final manuscript.
